# Hypopituitarism is associated with lower oxytocin concentrations and reduced empathic ability

**DOI:** 10.1007/s12020-017-1332-3

**Published:** 2017-06-08

**Authors:** Katie Daughters, Antony S. R. Manstead, D. Aled Rees

**Affiliations:** 1 School of Psychology, Cardiff University, Cardiff, CF10 3AT UK; 2 Neuroscience and Mental Health Research Institute, School of Medicine, Cardiff University, Cardiff, CF24 4HQ UK

**Keywords:** Central diabetes insipidus, Hypopituitarism, Oxytocin, Empathy

## Abstract

**Purpose:**

Central diabetes insipidus is characterised by arginine vasopressin deficiency. Oxytocin is structurally related to vasopressin and is synthesised in the same hypothalamic nuclei, thus we hypothesised that patients with acquired central diabetes insipidus and anterior hypopituitarism would display an oxytocin deficiency. Moreover, psychological research has demonstrated that oxytocin influences social and emotional behaviours, particularly empathic behaviour. We therefore further hypothesised that central diabetes insipidus patients would perform worse on empathy-related tasks, compared to age-matched and gender-matched clinical control (clinical control-isolated anterior hypopituitarism) and healthy control groups.

**Method:**

Fifty-six participants (age 46.54 ± 16.30 yrs; central diabetes insipidus: *n* = 20, 8 males; clinical control: *n* = 15, 6 males; healthy control: *n* = 20, 7 males) provided two saliva samples which were analysed for oxytocin and completed two empathy tasks.

**Results:**

Hypopituitary patients (both central diabetes insipidus and clinical control groups) had significantly lower oxytocin concentrations compared to healthy control participants. Hypopituitary patients also performed significantly worse on both the reading the mind in the eyes task and the facial expression recognition task compared to healthy control participants. Regression analyses further revealed that central diabetes insipidus patients’ oxytocin concentrations significantly predicted their performance on easy items of the reading the mind in the eyes task.

**Conclusions:**

Hypopituitarism may therefore be associated with reduced oxytocin concentrations and impaired empathic ability. While further studies are needed to replicate these findings, our data suggest that oxytocin replacement may offer a therapeutic approach to improve psychological well-being in patients with hypopituitarism.

## Introduction

Central diabetes insipidus (CDI) is an endocrine disorder with an estimated prevalence of 1 in 25000 [1]. CDI is characterised by a deficiency in arginine vasopressin (AVP), which occurs due to a significant loss (>80%) of function in hypothalamic neurons responsible for AVP synthesis [1]. AVP is the sister peptide of oxytocin (OT): they differ in structure by just two amino acids [2] and are produced in the paraventricular and supraoptic nuclei of the hypothalamus, where they are transported to the posterior pituitary gland for release into the peripheral circulation [3]. It seems likely therefore that patients with acquired CDI, as a result of pituitary surgery, will also present with a deficiency in OT.

OT is known to initiate uterine contractions during parturition and to initiate the milk let-down reflex during lactation. There is currently no recognised endocrine phenotype associated with OT deficiency. However, psychological research has shown that OT plays an important role in a range of social behaviours including increasing an individual’s ability to identify emotional expressions [4, 5], increasing the speed at which an individual accurately identifies emotional expressions [6], and regulating prosocial behaviour [7, 8]. As this line of research has developed, so has the interest in the therapeutic potential of OT to treat a range of psychological disorders that are characterised by deficits in these social behaviours. Indeed research has shown that several of these disorders (autism spectrum disorder [9], attention deficit hyperactivity disorder [10], and depression [11]) are associated with disrupted OT systems, and that intranasal OT administration can attenuate (to some extent) the characteristic empathy deficits [12, 13]. Thus prior research has shown that specific psychological disorders show a deficit in cognitive empathy, and that cognitive empathy can be moderated by OT.

The only study (to our knowledge) to investigate the presence of an OT deficiency in a patient group found that patients with childhood-onset craniopharyngiomas who had grade 1 lesions to the hypothalamus as a result of surgical resection had significantly lower OT concentrations compared to those with grade 2 lesions and those with no hypothalamic involvement [14]. The same research group went on to administer OT in ten of their patients [15], finding that grade 1 lesion patients’ performance on an empathy task improved after OT administration. The authors concluded that that it was important to consider OT with regard to potential behavioural pathologies in patients with craniopharyngiomas, specifically that OT may have beneficial effects on cognitive empathy.

The present study is a logical extension of this research: to investigate whether patients with acquired CDI would present with lower salivary OT concentrations, compared to a clinical and a healthy control (HC) group, and if so, whether this would have an effect on their emotion recognition skills.

## Materials and methods

### Participants and ethics

Fifty-five adults were recruited from the Endocrinology clinic at the University Hospital Wales into one of three groups: the CDI group, the clinical control (CC) group, and the HC group. The diagnosis of CDI and anterior hypopituitarism (HP) was established as per routine clinical practice by the water deprivation test, and a combination of basal and dynamic tests of anterior pituitary function, respectively. Inclusion criteria included documented biochemical evidence of anterior HP and CDI, if present. Participants were also required to receive full hormone replacement therapy for at least 3 months prior to study entry, while those with a history of functioning pituitary adenomas (acromegaly, prolactinoma or Cushing’s disease) were included only if in biochemical remission. CC patients were recruited using the same inclusion criteria, but were excluded if there was a history of partial or transient CDI. All participants in the CDI group had acquired CDI as a result of transsphenoidal pituitary surgery. Participants were excluded from the study if they were under 18 years of age, pregnant or breastfeeding, or if they were unable to comply fully with the protocol and study instructions. Participants were also excluded if they presented with any concomitant condition which could compromise the study objectives and/or preclude the protocol-defined procedures (e.g., psychiatric disorders). Finally, both the CC and HC participants were matched by age and gender to CDI patients; a one-way analysis of variance (ANOVA) revealed that there was no significant difference in age between the three groups, and there was a similar gender distribution between groups (see Table 1 for details).

**Table 1:** Clinical characteristics of the CDI and CC patients

		CDI (*n*)	CC (*n*)	HC (*n*)	*p*-value
Age (years)		44.05 ± 3.6	51.69 ± 4.4	45.31 ± 3.8	.382
Gender		8 male	6 male	7 male	
		12 female	9 female	13 female	
BMI (kg/m^2^)		32.72 ± 1.5	33.22 ± 1.7	27.66 ± 1.6	.031
Hypopituitarism	Partial hypopituitarism	8	12	N/A	
	Panhypopituitarism	7	3		
	Eupituitary	5	0		
Tumour type	Craniopharyngioma	9	1	N/A	
	Non-functioning pituitary adenoma	2	5		
	Somatotroph adenoma	2	3		
	Prolactinoma	2	2		
	Mammo-somatotroph adenoma	1	1		
	Corticotroph adenoma	0	2		
	Other	4	1		
Hormone replacement	Desmopressin	20	0	N/A	
	Hydrocortisone	11	10		
	Thyroxine	14	8		
	Growth hormone	2	2		
	Testosterone/oestrogen	8	3		

The study protocol was approved by Cardiff University (study sponsor), the Research and Development Office at the Cardiff and Vale University Health Board and by the Cambridge Central Research Ethics Committee (15/CMC/6297). All participants provided written informed consent, were fully debriefed at the end of the study, and financially compensated for their participation.

### Materials

#### Reading the mind in the eyes (RMET) task

**Fig. 1:**
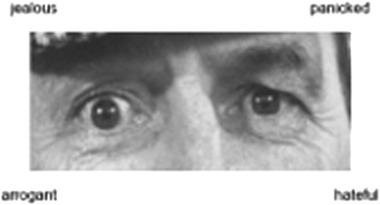
Example stimulus from the reading the mind in the eyes test

The RMET is a pre-existing validated measure of cognitive empathy created by Baron-Cohen et al. [16]. Participants are presented with 36 facial expressions displaying just the eye region of the face, and are therefore only able to use the eyes to infer the mental state of the actor (see Fig. 1). The faces included male and female actors. For each face, there are four response options and participants are asked to select the word that they feel best describes the face. Participants are instructed to work through the task at their own pace, and could refer to a definition list of the words at any time. A percentage of each participant’s total number of correct responses was calculated, in addition to two subscale scores, one for easy and one for difficult items [4].

#### Facial emotion recognition (FER) task

**Fig. 2:**
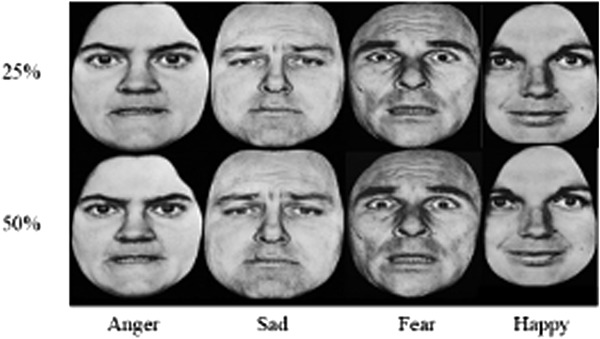
Example stimuli of the FER

A modified version of the FER task [12] was used to assess participants’ accuracy in identifying facial expressions of emotion. Participants saw male and female faces from the Ekman, Friesen [17] series, representing four ‘basic’ emotions (happiness, anger, fear and sadness) and neutral faces. Participants saw each expression six times at four different intensities (25, 50, 75, and 100%; differing intensities were created by morphing each expression with the actors’ neutral face), seeing a total of 96 faces (see Fig. 2). For each face, participants were asked “What emotion is this person showing?” and asked to select the number corresponding to that emotion (1 = happy, 2 = anger, 3 = fear, 4 = sad, 5 = neutral). Participants were instructed to work through the task at their own pace. A percentage of each participant’s correct responses was calculated. An error bias score was also calculated for each expression by summing the number of times participants incorrectly identified a face as expressing a particular emotion (e.g., all the times a participant reported fear, when the expression was another emotion).

#### Personality measures

The Interpersonal Reactivity Index (IRI) [18] is a well-established questionnaire, containing four subscales (empathic concern, fantasy, personal distress and perspective taking). A mean for each subscale is calculated providing a measure of participants’ trait empathy. All subscales obtained satisfactory internal consistency (empathic concern: *α* = .682; fantasy: *α* = .722; personal distress: *α* = .767; perspective taking: *α* = .814).

The Autism Quotient short version (AQ-S; [19]) is an adapted 28-item version of the original 50-item Autism Quotient [20], and provides a measure of participants’ tendencies to demonstrate autistic-like traits. Items relate to five subscales: social skills, mind reading, restricted and repetitive behaviour, imagination, and attention to detail. A mean for each subscale is created. All subscales apart from the restricted and repetitive behaviour subscale obtained satisfactory internal consistency (social skills: *α* = .752; mind reading: *α* = .702; restricted and repetitive behaviour: *α* = .429; imagination: *α* = .629; attention to detail: *α* = .706).

The Relationship Structure Questionnaire (ECR-RS) [21] is a previously validated questionnaire containing nine items about the participant’s relationship with their mother and father. A mean is calculated for each parent, providing a measure of participants’ attachment style. Both scales obtained satisfactory internal consistency (mother: *α* = .876; father: *α* = .890).

#### OT sampling and analysis

We chose to measure OT via saliva because (a) previous research has demonstrated saliva is a valid medium for measuring OT [14, 15, 22], (b) it is suggested that saliva has lower rates of non-specific binding compared to plasma samples [23], and (c) importantly, this is a non-invasive method. Passive drool saliva samples were therefore collected in pre-chilled tubes, stored on ice during the study, and frozen in a −80 °C freezer immediately after the second saliva sample was provided. Samples were then centrifuged, lyophilised and analysed in accordance with the manufacturer’s protocol, with an overnight incubation time of 19 h [24]. Full details regarding sampling and analysis are provided in [22]. Importantly, cross-reactivity with AVP is <0.02% (based on values provided by the manufacturer), thereby providing confidence that despite the structural similarity of vasopressin and OT, the results obtained would not be artificially high in the CDI group as a result of their desmopressin medication. The present study obtained intra-assay and inter-assay coefficients of <4% and 10.8–15.2%, respectively. All participants’ OT concentrations are provided in the Supplementary Materials.

#### Procedure

Participants were instructed to abstain from alcohol for 24 h and caffeine for an hour prior to testing. Participants were only allowed to drink water during the study and if any food had been consumed before the start of a session, participants were asked to rinse their mouths thoroughly before any saliva samples were taken. All testing was carried out between 09:00 and 12:00 in order to control for any hormonal circadian rhythms.

On arrival, participants’ height and weight were measured in order that their body mass index (BMI) could be calculated. After a brief period (approximately 10 min) of acclimatisation to the testing facility, participants were asked to provide their first saliva sample before completing either the RMET or FER task. Given the similarity of these tasks, the order in which they were presented was counterbalanced to control for any learning effects that might arise from the first task. Participants then completed the personality measure questionnaires before providing their second saliva sample, approximately 30 min later. After this, participants completed a behavioural task that will not be discussed here, before completing either the RMET or FER task according to their counterbalance condition. Participants were fully debriefed before leaving.

### Data analysis

#### Saliva data

An analysis of covariance (ANCOVA) was conducted to assess the effect of group on participants’ OT concentrations, while controlling for age, medication and BMI. A priori power calculations were based on our recent study in which salivary OT concentrations were assessed in male undergraduates [22], in whom we found a mean OT concentration of 46 pg/ml with a standard deviation of 33 pg/ml. Accordingly, recruiting 20 participants in each group would allow us to detect a 21 pg/ml standard deviation with 80% power at the 5% alpha level.

#### Personality data

Three ANOVAs were carried out to assess whether there were any group differences in each of the self-reported personality measures (any main effects of subscale or interactions were not the primary interest of the study and therefore only reported in Supplementary Materials).

#### Empathy data

An ANCOVA was carried out to investigate any differences in total RMET scores between groups, while controlling for age and gender. To investigate the relationship between RMET performance on easy and difficult items and OT concentrations, three multiple regression models were run in order to investigate group differences.

A mixed ANCOVA was carried out to assess the influence of different facial expressions, expression intensity, and group on participants’ accuracy during the FER. This analysis was repeated using participants’ OT concentrations as a covariate to identify whether OT concentrations were responsible for any observed group effects. Finally, a mixed ANOVA was carried out to assess whether there was an effect of any propensity of participants in a given group to over-report emotions during the FER, subsequently termed ‘error bias’.

All reported analyses control for age and gender as covariates, although age and gender were not significant covariates in any of these analyses.

## Results

### Descriptives

Table 1 summarises the medical characteristics of the CDI and CC clinical groups. By design, the majority (75%) of CDI patients had concomitant partial or pan-hypopituitarism. Eighty percent of CC patients had partial anterior HP, while the remainder were panhypopituitary. The range of tumours removed during surgery is broadly reflective of the diverse aetiology of both CDI and HP: 45% of CDI patients had surgery for craniopharyngioma, a common cause of CDI, compared to just one CC patient; 25% of CDI patients had a history of tumours producing either growth hormone, prolactin, or both, compared to 40% of CC patients; and 10% of CDI patients had non-functioning pituitary adenomas, compared to 33% of CC patients.

The age at which patients underwent surgery did not differ significantly between clinical groups, *t*(31) = −1.65, *p* = .109, and ranged from 2 to 72 years of age; the mean age at which patients underwent surgery was 36 years of age.

Finally, a two-way ANOVA revealed a significant difference in BMI between groups, *F*(2, 48) = 3.729, *p* = .031, $\eta _p^2$ = .134 (see Table 1 for details).

### OT analysis

A 3 (Group: CDI vs. CC vs. HC; between-subjects) × 2 (samples: 1 vs. 2; within- subjects) mixed ANCOVA revealed a trend towards a significant main effect of group on OT concentrations, *F*(2, 52) = 2.567, *p* = .086, $\eta _p^2$ = .090. A follow-up ANCOVA was carried out in which the CDI and CC groups were combined into one HP group. This analysis was deemed appropriate because (i) there was a similarity between CDI and CC patients in OT concentrations; (ii) there was also a similarity between the CDI and CC patients in empathy performance, as reported below; (iii) the original analysis only achieved 49% power.

Normality analysis revealed an outlier in the HP group which was removed, and also that the data did not meet the assumption of normality. A log transformation was carried out, after which the transformed values met the assumption of normality. All statistical analyses reported below were carried out on the transformed data. For ease of interpretation, untransformed means and standard errors are reported.

**Fig. 3:**
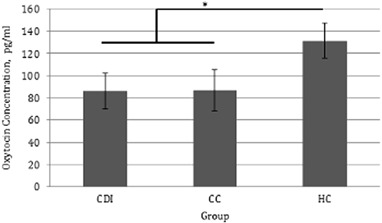
Average oxytocin concentrations for hypopituitary patients and healthy control participants (±SE)

There was a significant main effect of group, *F*(1, 46) = 4.922, *p* = .031, $\eta _p^2$ = .097, HP patients having significantly lower OT concentrations compared to HC participants (Fig. 3). Replicating the findings of the previous analysis, there was no main effect of sample, *F*(1, 46) = .193, *p* = .662, $\eta _p^2$ = .004, no interaction, *F*(1, 46) = .082, *p* = .776, $\eta _p^2$ = .002.

### Personality measures

A 4 (IRI subscale: empathic concern vs. fantasy vs. perspective taking vs. personal distress; within-subjects) × 3 (Group: CDI vs. CC vs. HC; between-subjects) mixed ANOVA revealed that CC (*M* = 2.195, SE = .111) and CDI (*M* = 2.220, SE = .102) patients had significantly lower trait empathy scores compared to HC (*M* = 2.538, SE = .096) participants, *F*(2, 50) = 3.639, *p* = .033, $\eta _p^2$ = .127.

A 4 (AQ-S Subscale: social skills vs. mind reading vs. imagination vs. attention to detail; within-subjects) × 3 (Group: CDI vs. CC vs. HC; between-subjects) mixed ANOVA revealed that CC patients (*M* = 2.502, SE = .095) had significantly lower AQ-S scores compared to HC (*M* = 2.952, SE = .085) participants, *F*(2, 51) = 6.269, *p* = .004, $\eta _p^2$ = .197. There was no difference between CDI patients (*M* = 2.710, SE = .090) and HP patients or HC participants.

A 2 (ECR-RS Subscale: mother vs. father; within-subjects) × 3 (Group: CDI vs. CC vs. HC; between-subjects) mixed ANOVA revealed no main effects or interaction.

### Empathy analysis

#### RMET

**Fig. 4:**
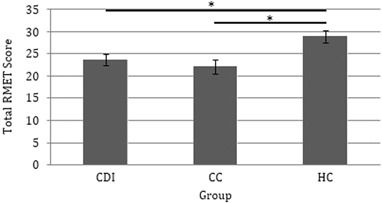
RMET score as a function of clinical group (±SE)

A one-way ANCOVA was carried out to investigate the effect of group (CDI vs. CC vs. HC) on total RMET scores. CDI and CC patients had significantly lower RMET scores compared to HC participants (Fig. 4), but there was no difference between CDI and CC scores, *F*(2, 42) = 5.557, *p* = .007, $\eta _p^2$ = .209.

Regression models conducted within each participant group revealed that OT response predicted CDI participants’ performance on easy items (see Table 2).

**Table 2:** OT response specifically predicts CDI patients’ performance for easy items, not difficult items, of the RMET

Scale	Group	_*R*_2	F	d*f*	*p*	β
Easy	CDI	.277	6.513	(1,17)	.021	.526
	CC	.214	2.730	(1,10)	.129	.463
	HC	.022	.396	(1,18)	.537	.147
Difficult	CDI	.062	1.128	(1,17)	.303	.249
	CC	.093	1.025	(1,10)	.335	.305
	HC	.080	1.563	(1,18)	.227	.283

#### FER

A mixed 4 (Emotion: happy vs. sad vs. fear vs. anger; within-subjects) × 4 (intensity: 25 vs. 50 vs. 75 vs. 100; within-subjects)  × 3 (Group: CDI vs. CC vs. HC; between-subjects) ANOVA revealed that CC patients (*M* = 61.122, SE = 2.103) had significantly lower scores than HC participants (*M* = 70.590, SE = 1.870), but there was no difference between CDI patients (*M* = 65.949, SE = 1.840) and CC or HC participants, *F*(2, 44) = 5.680, *p* = .006, $\eta _p^2$ = .205.

There was a significant main effect of emotion and intensity and a significant interaction, full details of which are provided in the Supplemental Materials.

**Fig. 5:**
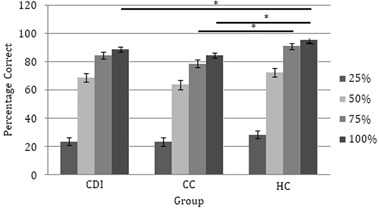
FER performance as a function of intensity and clinical group (±SE)

Simple effects analyses were carried out to assess the effect of group on recognition of expressions varying in emotion and intensity. Group had no effect on recognition of happy, sad, or angry expressions, but CC patients (*M* = 55.833, SE = 2.986) correctly identified significantly fewer fearful expressions compared to HC participants (*M* = 67.292, SE = 2.586), *F*(2, 51) = 4.331, *p* = .018, $\eta _p^2$ = .145; there was no difference between CDI and CC patients. Unexpectedly, group also had a significant effect on high intensity facial expressions: 100%, *F*(2, 51) = 11.491, *p* < .001, $\eta _p^2$ = .311; 75%, *F*(2, 51) = 6.308, *p* = .004, $\eta _p^2$ .198. At 100% intensity, both CC and CDI patients identified fewer expressions correctly compared to HC participants (there was no difference between CDI and CC patients). At 75% intensity, CC patients correctly identified fewer expressions compared to HC participants. There was no effect of group on recognition of low intensity expressions. The relevant means and standard errors are presented in Fig. 5.

Finally, a 4 (emotion: happy vs. sad vs. fear vs. anger; within-subjects)  × 3 (Group: CDI vs. CC vs. HC; between-subjects) mixed ANOVA revealed that CDI (*M* = 3.369, SE = .392) and CC (*M* = 3.714, SE = .445) patients had larger error biases compared to HC (*M* = 2.338, SE = .372) participants, *F*(2, 49) = 3.962, *p* = .025, $\eta _p^2$ = .139. Simple effects analysis revealed a significant difference between the groups for fear bias, *F*(2, 49) = 4.002, *p* = .025,  = .140, and anger bias, *F*(2, 49) = 5.559, *p* = .007, $\eta _p^2$ = .185: CC patients had a greater fear bias (*M* = 5.429, SE = .745) compared to HC participants (*M* = 2.750, SE = .624), and both CDI (*M* = 4.500, SE = .596) and CC (*M* = 5.000, SE = .676) patients had a greater anger bias compared to HC participants (*M* = 2.350, SE = .565).

OT was not a significant covariate of participants’ FER accuracy or of their error bias. The original main effects and interaction remained significant when controlling for OT.

## Discussion

We investigated whether patients with acquired CDI, characterised by a deficiency in AVP, would also demonstrate a deficit in OT, AVP’s ‘sister’ peptide, and if so, whether they would also demonstrate a deficit in cognitive empathy (compared to a CC and HC participants). The results are consistent with these hypotheses: HP patients had significantly lower OT concentrations compared to HC participants, and also scored significantly lower on the RMET and FER compared to HC participants. However, the prediction that CDI patients would have significantly lower OT concentrations than CC patients was not supported. This may reflect an influence of anterior pituitary hormones on OT secretion or an unintentional disruption to hypothalamic-pituitary neurones responsible for OT synthesis during surgery, an explanation that was supported in previous research [14]. Because all CDI patients had undergone transsphenoidal surgery the likelihood of hypothalamic lesions accounting for these findings is low; it is beyond the scope of the present study to quantify hypothalamic involvement on the basis of scan data. CDI and CC groups were subsequently collapsed into a new HP group which was deemed justifiable because of the comparable OT concentrations demonstrated in the original analysis; the phenotypic similarity during the empathy tasks; and due to the fact that the three group ANOVA was significantly underpowered.

Despite HP patients presenting with lower OT concentrations, their concentrations were not especially low. By definition, patients with CDI had acquired sufficient disruption to the hypothalamic–pituitary neurones to impair AVP release; it could therefore be reasonably anticipated that they would exhibit an equivalent and significant decrease in OT release. Although these results may potentially be explained as a result of the similarity and potential interaction between AVP and OT systems [25, 26], or as a result of dendritic release of OT in other regions of the brain [27], it should be acknowledged that HC concentrations were also higher than expected. This may relate to issues of noise in the ELISA method. While we accept this is a limitation of the study, and are therefore cautious about drawing conclusions on the basis of the absolute values observed, there is no reason (to our knowledge) why noise should vary significantly between groups. Thus the significant difference between groups should not be dismissed.

HP patients also presented with a significant empathy deficit. Initial analyses of self-report measures found that HP patients had significantly lower trait empathy compared to HC participants. This was supported by experimental data showing that CDI patients scored significantly lower on the RMET compared to HC participants, and participants’ OT concentrations significantly predicted their performance on the easy items of the RMET: the lower their OT concentrations, the lower their score. On the FER task, CC patients had significantly lower overall scores than HC participants, which was driven by a specific difficultly in identifying fearful facial expressions. The scores of CDI patients tended to fall between the CC and HC scores. Both CDI and CC patients were poorer at identifying high intensity expressions (compared to HC participants), which one would expect to be easier to identify. Both clinical groups also demonstrated a greater error bias, compared to HC participants; CC patients demonstrated a fear bias, in that CC patients tended to over report fear when presented with other emotions, and both CC and CDI patients had an anger bias. However, participants’ OT concentrations were not related to their FER performance.

These results support findings reported by Hoffmann et al. [15] showing that grade 1 lesion craniopharyngioma patients performed significantly better on an empathy task after OT administration, providing support for the beneficial role of OT in the emotional behaviour of medical patient groups; and that this increase was driven by an improvement at identifying negative emotions.

In the present study, a further novel finding was that CC patients were significantly worse at recognising fearful facial expressions and while showing also a tendency to report that other facial expressions depicted fear (when they did not) is especially noteworthy. Previous research has demonstrated that clinical groups who over-report negative (in particular fearful) expressions may be at greater risk of developing mood disorders [28, 29]. The reverse may also be true: patients with mood disorders display a bias towards negative emotions [30]. Thus an important avenue for future research is to investigate whether patients with HP are also at an increased risk of mood disorders, especially in view of the fact that previous research has shown that deficits in empathic ability have a significant impact on social relationships [31, 32] and mood [33, 34]. Consistent with this reasoning, a previous study [35] demonstrated that adults who had childhood-onset multiple pituitary hormone deficiencies have a significantly lower quality of life, compared to their physically healthy peers. Moreover, the fact that CC patients presented with low OT and a negative emotional bias complements the approach/withdrawal hypothesis of OT [36] suggesting that these patients may benefit from OT administration.

There are some limitations of the study that should be acknowledged. We sought to recruit 20 participants for each group; however, we were only able to recruit 15 CC patients who were appropriately age- and gender-matched. Consequently, the results pertaining to CC patients need to be treated with some caution. A future study should therefore aim to achieve a larger sample size to confirm the generalisability of the present results. Such a study may also wish to match participants on intellectual ability to control for this variable. In addition, although we were specifically interested in the effects of OT, we acknowledge that hormones do not act in isolation and it may be the case that other hormones/neurotransmitters could explain additional variance between our groups. Importantly, a strength of the study is the age range of participants (22–74). This, in combination with the fact that age was not a significant covariate, suggests that age does not moderate any effects of group or OT on cognitive empathy. Finally, it is important to note that we did not administer intranasal OT, and thus cannot draw inferences about causality.

In conclusion, the present study found that HP patients had significantly lower OT concentrations than HC participants, and also had significant deficits in cognitive empathy. Further studies are needed to replicate both these and previous findings [15], in order to establish whether OT supplementation can reverse these deficits with a view to improving psychological well-being.

## Electronic supplementary material

Supplementary Material

## References

[1] Ball S (2005). Diabetes insipidus. Medicine.

[2] Brownstein MJ (1983). Biosynthesis of vasopressin and oxytocin. Annu. Rev. Physiol..

[3] Swaab D, Nijveldt F, Pool C (1975). Distribution of oxytocin and vasopressin in the rat supraoptic and paraventricular nucleus. J. Endocrinol..

[4] Domes G, Heinrichs M, Michel A, Berger C, Herpertz SC (2007). Oxytocin improves “mind-reading” in humans. Biol. Psychiatry.

[5] Shahrestani S, Kemp AH, Guastella AJ (2013). The impact of a single administration of intranasal oxytocin on the recognition of basic emotions in humans: a meta-analysis. Neuropsychopharmacology.

[6] Hubble Kelly, Daughters Katie, Manstead Antony S.R., Rees Aled, Thapar Anita, van Goozen Stephanie H.M. (2016). Oxytocin Reduces Face Processing Time but Leaves Recognition Accuracy and Eye-Gaze Unaffected. Journal of the International Neuropsychological Society.

[7] Ten Velden Femke S., Daughters Katie, De Dreu Carsten K.W. (2017). Oxytocin promotes intuitive rather than deliberated cooperation with the in-group. Hormones and Behavior.

[8] De Dreu CK (2012). Oxytocin modulates cooperation within and competition between groups: an integrative review and research agenda. Horm. Behav..

[9] Husarova VM, Lakatosova S, Pivovarciova A, Babinska K, Bakos J, Durdiakova J, Kubranska A, Ondrejka I, Ostatnikova D (2016). Plasma oxytocin in children with autism and its correlations with behavioral parameters in children and parents. Psychiatry Invest..

[10] Demirci E, Ozmen S, Kilic E, Oztop DB (2016). The relationship between aggression, empathy skills and serum oxytocin levels in male children and adolescents with attention deficit and hyperactivity disorder. Behav. Pharmacol..

[11] Jobst A, Sabass L, Palagyi A, Bauriedl-Schmidt C, Mauer MC, Sarubin N, Buchheim A, Renneberg B, Falkai P, Zill P (2015). Effects of social exclusion on emotions and oxytocin and cortisol levels in patients with chronic depression. J. Psychiatr. Res..

[12] Bowen KL, Morgan JE, Moore SC, van Goozen SH (2014). Young offenders’ emotion recognition dysfunction across emotion intensities: explaining variation using psychopathic traits, conduct disorder and offense severity. J. Psychopathol. Behav. Assess..

[13] Guastella AJ, Einfeld SL, Gray KM, Rinehart NJ, Tonge BJ, Lambert TJ, Hickie IB (2010). Intranasal oxytocin improves emotion recognition for youth with autism spectrum disorders. Biol. Psychiatry.

[14] Daubenbüchel Anna M. M., Hoffmann Anika, Eveslage Maria, Özyurt Jale, Lohle Kristin, Reichel Julia, Thiel Christiane M., Martens Henri, Geenen Vincent, Müller Hermann L. (2016). Oxytocin in survivors of childhood-onset craniopharyngioma. Endocrine.

[15] Hoffmann Anika, Özyurt Jale, Lohle Kristin, Reichel Julia, Thiel Christiane M., Müller Hermann L. (2017). First experiences with neuropsychological effects of oxytocin administration in childhood-onset craniopharyngioma. Endocrine.

[16] Baron-Cohen S, Wheelwright S, Hill J, Raste Y, Plumb I (2001). The “reading the mind in the eyes” test revised version: a study with normal adults, and adults with Asperger syndrome or high‐functioning autism. J. Child Psychol. Psychiatry.

[17] Ekman P, Friesen WV (1975). Pictures of Facial Affect.

[18] Davis MH (1983). Measuring individual differences in empathy: evidence for a multidimensional approach. J. Pers. Soc. Psychol..

[19] Kloosterman PH, Keefer KV, Kelley EA, Summerfeldt LJ, Parker JD (2011). Evaluation of the factor structure of the autism-spectrum quotient. Pers. Individ. Dif..

[20] Baron-Cohen S, Wheelwright S, Skinner R, Martin J, Clubley E (2001). The autism-spectrum quotient (AQ): evidence from asperger syndrome/high-functioning autism, males and females, scientists and mathematicians. J. Autism Dev. Disord..

[21] Fraley RC, Heffernan ME, Vicary AM, Brumbaugh CC (2011). The experiences in close relationships—relationship structures questionnaire: a method for assessing attachment orientations across relationships. Psychol. Assess..

[22] Daughters K, Manstead AS, Hubble K, Rees A, Thapar A, Goozen HM (2015). Salivary oxytocin concentrations in males following intranasal administration of oxytocin: A double-blind, cross-over study. PLoS One.

[23] G. Leng, N. Sabatier, Measuring oxytocin and vasopressin: Bioassays, immunoassays and random numbers. J. Neuroendocrinol. (2016). doi:10.1111/jne.12413 10.1111/jne.12413 PMC5096068 27467712

[24] Product Manual: Oxytocin ELISA kit. (2013) http://static.enzolifesciences.com/fileadmin/files/manual/ADI-901-153A_insert.pdf

[25] Bernal A, Mahía J, Puerto A (2016). Animal models of central diabetes insipidus: Human relevance of acquired beyond hereditary syndromes and the role of oxytocin. Neurosci. Biobehav. Rev..

[26] Weisman O, Schneiderman I, Zagoory-Sharon O, Feldman R (2013). Salivary vasopressin increases following intranasal oxytocin administration. Peptides.

[27] Ludwig M, Leng G (2006). Dendritic peptide release and peptide-dependent behaviours. Nat. Rev. Neurosci..

[28] Beck AT (2008). The evolution of the cognitive model of depression and its neurobiological correlates. Am. J. Psychiatry.

[29] Beck AT (1979). Cognitive therapy and the emotional disorders.

[30] Leppänen JM (2006). Emotional information processing in mood disorders: a review of behavioral and neuroimaging findings. Curr. Opin. Psychiatry.

[31] Stephan WG, Finlay K (1999). The role of empathy in improving intergroup relations. J. Soc. Issues.

[32] Fischer AH, Manstead AS (2008). Social functions of emotion. Handb. Emot..

[33] Van Kleef GA, De Dreu CK, Manstead AS (2010). An interpersonal approach to emotion in social decision making: the emotions as social information model. Adv. Exp. Soc. Psychol..

[34] Van Kleef GA (2009). How emotions regulate social life the emotions as social information (EASI) model. Curr. Dir. Psychol. Sci..

[35] Kao K-T, Stargatt R, Zacharin M (2015). Adult quality of life and psychosocial outcomes of childhood onset hypopituitarism. Horm. Res. Paediatr..

[36] Kemp AH, Guastella AJ (2011). The role of oxytocin in human affect: a novel hypothesis. Curr. Dir. Psychol. Sci..

