# Supplementary material for: Hypopituitarism is associated with lower oxytocin concentrations and reduced empathic ability
Source: Endocrine. 2017 Jun 8;57(1):166–74. doi: 10.1007/s12020-017-1332-3 (PMC5486505; doi:10.1007/s12020-017-1332-3)
Supplement: Supplementary file 1 — Supplementary Material [file 12020_2017_1332_MOESM1_ESM.docx]

# Supplementary Material

Personality Measures

A 4 (IRI subscale: Empathic Concern vs Fantasy vs Perspective Taking vs Personal Distress; within-subjects) x 3 (Group: CDI vs HP vs HC; between-subjects) mixed ANOVA was carried out. There was a significant main effect of IRI subscale, *F*(3, 150) = 33.485, *p* < .001, η^2^_p_ = .401, such that participants scored more highly on the Empathic Concern subscale (*M* = 3.056, *SE* = .082) compared to Fantasy (*M* = 2.070, *SE* = .106), Perspective Taking (*M* = 2.389, *SE* = .103) and Personal Distress (*M* = 1.756, *SE* = .116); there was no difference between Fantasy and Perspective Taking scores; and Fantasy scores were higher than Personal Distress scores. There was also a main effect of group, *F*(2, 50) = 3.639, *p* = .033, η^2^_p_ = .127, such that HP patients (*M* = 2.195, *SE* = .111) and CDI patients (*M* = 2.220, *SE* = .102) had lower scores compared to HC participants (*M* = 2.538, *SE* = .096). Finally there was a significant interaction between group and subscale, *F*(4.867, 121.677) = 3.460, *p* = .006, η^2^_p_ = .122. Simple effects analysis revealed that there was no difference between groups on the Empathic Concern (*F*(2, 50) = 1.069, *p* = .351) or Personal Distress (*F*(2, 50) = .554, *p* = .578) subscales, however CDI patients scored significantly lower on the both Fantasy (*F*(2, 50) = 7.368, *p* = .002) and Perspective Taking (*F*(2, 50) = 4.812, *p* = .012) subscales compared to HC participants, while HP patients only scored significantly lower compared to HC participants on the Fantasy subscale (see Table SM.1).

*Table SM.1 - Means and standard errors relating to the group by IRI subscale interaction*

| **Group** | **IRI Subscale** | **Mean** | **SE** |
| --- | --- | --- | --- |
| **CDI** | Empathic Concern | 3.111 | .139 |
|  | Fantasy | 1.837 | .180 |
|  | Perspective Taking | 1.976 | .175 |
|  | Personal Distress | 1.921 | .198 |
| **HP** | Empathic Concern | 2.886 | .153 |
|  | Fantasy | 1.714 | .197 |
|  | Perspective Taking | 2.476 | .192 |
|  | Personal Distress | 1.705 | .217 |
| **HC** | Empathic Concern | 3.171 | .132 |
|  | Fantasy | 2.621 | .171 |
|  | Perspective Taking | 2.714 | .166 |
|  | Personal Distress | 1.643 | .188 |

A 4 (AQ-S Subscale: Social Skills vs Mind Reading vs Imagination vs Attention to Detail; within-subjects) x 3 (Group: CDI vs HP vs HC; between-subjects) mixed ANOVA was carried out. There was a main effect of subscale, *F*(3, 153) = 19.676, *p* < .001, η^2^_p_ = .278, such that participants scored more highly on the Imagination subscale (*M* = 3.118, *SE* = .085) compared to Social Skills (*M* = 2.758, *SE* = .086, Mind Reading (*M* = 2.769, *SE* = .083), and Attention to Detail (*M* = 2.241, *SE* = .097); there was no difference between Social Skills and the Mind Reading scores, but both scores were greater than the Attention to Detail subscale. There was a significant main effect of group, *F*(2, 51) = 6.269, *p* = .004, η^2^_p_ = .197, such that HP patients (*M* = 2.502, *SE* = .095) scored significantly lower compared to HC participants (*M* = 2.952, *SE* = .085), but there was no difference between CDI patients (*M* = 2.710, *SE* = .090) and HP patients or HC participants. There no significant interaction, *F*(5.033, 128.350) = 1.398, *p* = .229, η^2^_p_ = .052, however simple effects analysis did reveal a significant difference between groups on the Attention to Detail subscale, *F*(2, 51) = 7.083, *p* = .002, such that HP patients (*M* = 1.750, *SE* = .177) scored significantly lower, and CDI patients (*M* = 2.333, *SE* = .167) trended towards lower scores, compared to HC participants (*M* = 2.640, *SE* = .159).

FER

A mixed 4 (Emotion: happy vs sad vs fear vs anger; within-subjects) x 4 (Intensity: 25 vs 50 vs 75 vs 100; within-subjects) x 3 (Group: CDI vs HP vs HC; between-subjects) ANOVA was carried out. There was a significant main effect of emotion, *F*(2.34, 102.96) = 5.384, *p* < .004, η^2^_p_ = .109, reflecting the fact that more happy (*M* = 80.835, *SE* = 1.589) facial expressions were correctly identified, compared to sad (*M* = 60.993, *SE* = 2.696), fearful (*M* = 60.173, *SE* = 1.592) and angry (*M* = 61.547, *SE* = 1.947) expressions.

There was also a main effect of intensity, *F*(3, 132) = 42.107, *p* < .001, η^2^_p_ = .489, reflecting the fact that higher intensity expressions were more often identified correctly (100%: *M* = 88.666, *SE* = 1.010; 75%: *M* = 83.323, *SE* = 1.441; 50%: *M* = 67.067, *SE* = 1.837; 25%: *M* = 24.493, *SE* = 1.709). There was also a significant interaction between emotion and intensity, *F*(6.68, 293.75) = 2.496, *p* = .018, η^2^_p_ = .054. Bonferroni corrected pairwise comparisons revealed that more happy, fearful, and angry facial expressions were identified correctly at 50% intensity, compared to 25%, and at 75% compared to 50%; however, the difference in scores between 75% and 100% was not significant, although this apparent ceiling effect was not present for sad facial expressions (see Table SM.2).

*Table SM.2 - Means and standard errors relating to the emotion by intensity interaction*

| **Emotion** | **Intensity** | **Mean** | **SE** |
| --- | --- | --- | --- |
| **Happy** | 25% | 44.671 | 4.169 |
|  | 50% | 86.303 | 2.436 |
|  | 75% | 95.514 | 1.601 |
|  | 100% | 96.854 | 1.267 |
| **Sad** | 25% | 24.642 | 3.509 |
|  | 50% | 59.425 | 4.059 |
|  | 75% | 74.913 | 3.356 |
|  | 100% | 84.991 | 2.276 |
| **Fear** | 25% | 13.022 | 2.130 |
|  | 50% | 62.272 | 3.099 |
|  | 75% | 82.077 | 2.707 |
|  | 100% | 83.318 | 2.087 |
| **Anger** | 25% | 15.637 | 2.340 |
|  | 50% | 60.265 | 3.603 |
|  | 75% | 80.788 | 2.828 |
|  | 100% | 89.500 | 2.628 |

Finally a 4 (Emotion: happy vs sad vs fear vs anger; within-subjects) x 3 (Group: CDI vs HP vs HC; between-subjects) mixed ANOVA revealed a significant main effect of emotion on error bias, *F*(3, 147) = 12.025, *p* < .001, η^2^_p_ = .197; participants had a significantly lower bias towards happy (*M* = .819, *SE* = .192) compared to sadness (*M* = 4.315, *SE* = .747), fear (*M* = 3.950, *SE* = .354) and anger (*M* = 3.837, *SE* = .391). There was also a significant main effect of group, *F*(2, 49) = 3.962, *p* = .025, η^2^_p_ = .139, reflecting the fact that CDI (*M* = 3.369, *SE* = .392) and HP (*M* = 3.714, *SE* =.445) patients had larger error biases than HC participants did (*M* = 2.338, *SE* = .372). Although there was no significant interaction, *F*(3.743, 91.699) = 1.524, *p* = .205, η^2^_p_ = .059, simple effects analysis revealed a significant difference between the groups for fear bias, *F*(2, 49) = 4.002, *p* = .025, η^2^_p_ = .140, and anger bias, *F*(2, 49) = 5.559, *p* = .007, η^2^_p_ = .185: HP patients had a greater fear bias (*M* = 5.429, *SE* = .745) compared to HC participants (*M* = 2.750, *SE* = .624), and both CDI (*M* = 4.500, *SE* = .596) and HP (*M* = 5.000, SE = .676) patients had a greater anger bias compared to HC participants (*M* = 2.350, *SE* = .565). All other pairwise comparisons were non-significant, and there was no effect of group on happy bias, *F*(2, 49) = .671, *p* = .516, η^2^_p_ = .027, or sad bias, *F*(2,49) = .945, *p* = .396, η^2^_p_ = .037.

*Oxytocin Concentrations*

*Table SM.3 – Participants’ oxytocin concentrations across the session by group*

| Participant | Group | Saliva Sample 1 | Saliva Sample 2 |
| --- | --- | --- | --- |
| 1 | 1 | 109.26 | 118.02 |
| 2 | 1 | 71.48 | 48.91 |
| 3 | 2 | 88.37 | 88.37 |
| 4 | 1 | 63.26 | 58.56 |
| 5 | 1 | 80.25 | 92.44 |
| 6 | 1 | 19.88 | 12.36 |
| 7 | 2 | 57.44 | 87.24 |
| 8 | 1 | 54.21 | 48.29 |
| 9 | 1 | 98.58 | 84.48 |
| 10 | 1 | 90.67 | 176.96 |
| 11 | 3 | 42.19 | 60.86 |
| 12 | 2 | 39.31 | 47.98 |
| 13 | 2 | 39.82 | 32.83 |
| 14 | 3 | 71.48 | 71.02 |
| 15 | 1 | 79.73 | 55.63 |
| 16 | 2 | 33.47 | 36.39 |
| 17 | 1 | 83.4 | 58.94 |
| 18 | 3 | 106.69 | 71.82 |
| 19 | 1 | 63.07 | 79.97 |
| 20 | 2 | 93.68 | 128.56 |
| 21 | 1 | 34.06 | 36.04 |
| 22 | 3 | 55.38 | 92.1 |
| 23 | 2 | 121.5 | 114.82 |
| 24 | 3 | 89.54 | 66.74 |
| 25 | 1 | 47.01 | 59.6 |
| 26 | 3 | 164.87 | 184.6 |
| 27 | 3 | 37.71 | 29.74 |
| 28 | 2 | 211.42 | 165.8 |
| 29 | 3 | 372.07 | 314.04 |
| 30 | 1 | 28.26 | 69.43 |
| 31 | 1 | 283.66 | 158.47 |
| 32 | 1 | 320.07 | 246.28 |
| 33 | 3 | 314.04 | 182.52 |
| 34 | 1 | 26.56 | 35.43 |
| 35 | 2 | 52.04 | 122.88 |
| 36 | 3 | 66.2 | 289.43 |
| 37 | 3 | 39.38 | 61.61 |
| 38 | 2 | 72.72 | 57.02 |
| 39 | 3 | 56.71 | 53.07 |
| 40 | 3 | 76.85 | 41.62 |
| 41 | 3 | 305.87 | 270.86 |
| 42 | 3 | 176.03 | 105.3 |
| 43 | 2 | 50.78 | 61.61 |
| 44 | 1 | 151.63 | 76.42 |
| 45 | 3 | 77.27 | 76.85 |
| 46 | 2 | 155.02 | 172.18 |
| 47 | 3 | 62.64 | 60.26 |
| 48 | 2 | 43.5 | 36.85 |
| 49 | 1 | 40.48 | 41.62 |
| 50 | 2 | 91.2 | 138.8 |
| 51 | 2 | 85.83 | 70.35 |
| 52 | 3 | 120.89 | 136.52 |
| 53 | 3 | 223.23 | 222 |
| 54 | 3 | 225.4 | 183.53 |
| 55 | 1 | 76.07 | 64.92 |
